# An Ultra‐Flexible Neural Electrode with Bioelectromechanical Compatibility and Brain Micromotion Detection

**DOI:** 10.1002/adhm.202503101

**Published:** 2025-09-28

**Authors:** Donglei Chen, Yu Lu, Shuo Zhang, Wenqi Zhang, Zejie Yu, Shuideng Wang, Zhi Qu, Mingxing Cheng, Yiqing Yao, Deheng Wang, Zhan Yang, Lixin Dong

**Affiliations:** ^1^ Department of Biomedical Engineering City University of Hong Kong Hong Kong 999077 China; ^2^ Centre for Robotics and Automation City University of Hong Kong Shenzhen Research Institute Shenzhen 518057 China; ^3^ School of Integrative Medicine Shanghai University of Traditional Chinese Medicine Shanghai 201203 China; ^4^ School of Mechanical and Electrical Engineering Soochow University Suzhou 215137 China; ^5^ Jiangsu Key Laboratory of Embodied Intelligence Robot Technology Soochow University Suzhou 215137 China

**Keywords:** bioelectromechanical compatibility, brain micromotion detection, brain‐computer interface, neural electrode

## Abstract

Neural electrodes, as core components of brain‐computer interfaces(BCIs), face critical challenges in achieving stable mechanical coupling with brain tissue to ensure high‐quality signal acquisition. Current flexible electrodes, including semi‐invasive meningeal‐attached types and implantable cantilever designs, exhibit significant mechanical mismatches (elastic modulus 5–6 orders higher than brain tissue) due to material/structural limitations, leading to interfacial slippage. While thread‐like implants (e.g., Neuralink's electrodes) improve compliance via elongated structures, quantitative characterization of mechano‐bioelectric interactions remains unexplored. This study proposes a bioelectromechanical coupling strategy, emphasizing synchronized motion between the electrode and the brain tissue through exposed‐end deformation. A 4‐channel ultra‐flexible electrode (40 mm in length, 164 µm in width, and 3 µm in thickness) is optimized using finite‐element simulations and zero relative‐motion criteria, achieving an equivalent stiffness of 0.023 N m^−1^—matching brain tissue micromotion stiffness. A nanorobotic manipulator installed inside a scanning electron microscope(SEM) with an atomic force microscope(AFM) cantilever enabled precision characterization under the simulated displacement of 25 µm, revealing interfacial forces of 575 nN and piezoresistive sensitivities of 6.4 pA mm^−1^ (length) and 10.2 pA µm^−1^ (displacement). The dual‐functionality (signal acquisition and micromotion sensing) electrodes demonstrate breakthrough potential, establishing quantitative design standards for next‐generation bioelectronic implants.

## Graphical abstract

Neural electrodes face a mechanical mismatch with brain tissue. This study proposes a bioelectromechanical coupling strategy using an ultra‐flexible electrode designed for synchronized motion. Optimized to match brain tissue stiffness, it achieves dual signal acquisition and micromotion sensing, with characterized interfacial forces and piezoresistive sensitivity. This work establishes a quantitative design standard for next‐generation, multifunctional neural implants.

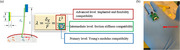

## 1 Introduction

**Figure 1:**
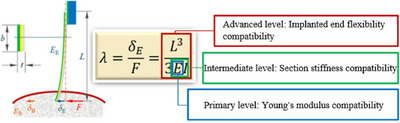
Three levels of mechanical properties match the principles between the neural electrode and brain tissue.

As the core component of brain‐computer interface (BCI) systems, neural electrodes serve dual functions of bioelectrical signal acquisition and transmission.^[^ ^1^, ^2^ ^]^ To achieve high‐quality electrophysiological signal acquisition and ensure long‐term stability of implanted devices, stable interfacial contact between electrode sampling sites and neural cells must be maintained.^[^ ^3^, ^4^ ^]^ Ideally, the electrode‐brain tissue interface should achieve a zero‐relativemotion mechanical coupling state. While current flexible electrode technologies conceptually align with this requirement, they face significant practical limitations. Meningeal‐adhered electrodes^[^ ^5^, ^6^, ^7^ ^]^ employ semi‐invasive flexible film structures and these electrodes conform to meningeal topology in the normal direction. However, their in‐plane stiffness is constrained by intrinsic material properties (elastic modulus remains 5–6 orders of magnitude higher than brain tissue), resulting in pronounced tangential mechanical mismatch and inevitable interfacial slippage. The compatibility is at the primary level in **Figure** **1**, and a perfect match is impossible to achieve. Implantable cantilever electrodes^[^ ^8^, ^9^, ^10^ ^]^ are designed to accommodate motion through structural bending. Their compliance enhancement is physically limited by multi‐channel integration requirements (minimum linewidth constraints) and microfabrication limitations (thickness controllability). Theoretical calculations show that even reducing the width of rectangular‐section electrodes by three orders of magnitude or the thickness by one order, their equivalent stiffness remains three orders of magnitude higher than that of brain tissue. Although this kind of compatibility has been improved in this way, it is also only at the intermediate level in Figure 1. For thread‐like implantable electrodes^[^ ^11^, ^12^, ^13^ ^]^ (e.g., Neuralink's sewing machine‐inspired design), their increased implantation length improves compliance. However, two critical theoretical gaps persist: the absence of quantitative mechanical matching characterization systems and systemic neglect of the mechanical coupling effects at exposed electrode terminals. This corresponds to the compatibility at the advanced level in Figure 1. Based on the above analysis, this study proposes a bioelectromechanical compatibility design philosophy, establishing a synchronized motion mechanism through exposed‐terminal deformation control. A multi‐scale research approach (mechanical modeling, finite element simulation, in situ electromechanical detection, and biomimetic brain tissue testing) was employed to elucidate full flexible matching mechanisms and develop electrode structural optimization criteria. The first is the structural optimization of the electrode. A single 4‐channel electrode prototype was developed under the zero relative‐motion criterion for implanted segments and brain tissue, with key parameters: 40 mm in length, 164 µm in width, and 3 µm in thickness, achieving an equivalent stiffness of 0.023 N m^−1^ (matching brain tissue micromotion stiffness). The second is the precision characterization of the electrode. A scanning electron microscope (SEM)‐atomic force microscope (AFM) cantilever integrated micro‐/nanorobotic manipulation system was constructed to simulate brain tissue‐equivalent micromotion (25 µm displacement), measure interfacial mechanical responses, and monitor dynamic impedance. This study transcends traditional flexible electrode design paradigms through two key advancements: establishment of quantitative design standards for electrode‐brain tissue mechanical compatibility and discovery of dual‐functional electrode properties (signal acquisition and micromotion sensing).

## 2 Simulation of the Ultra‐Flexible Neural Electrode

**Figure 2:**
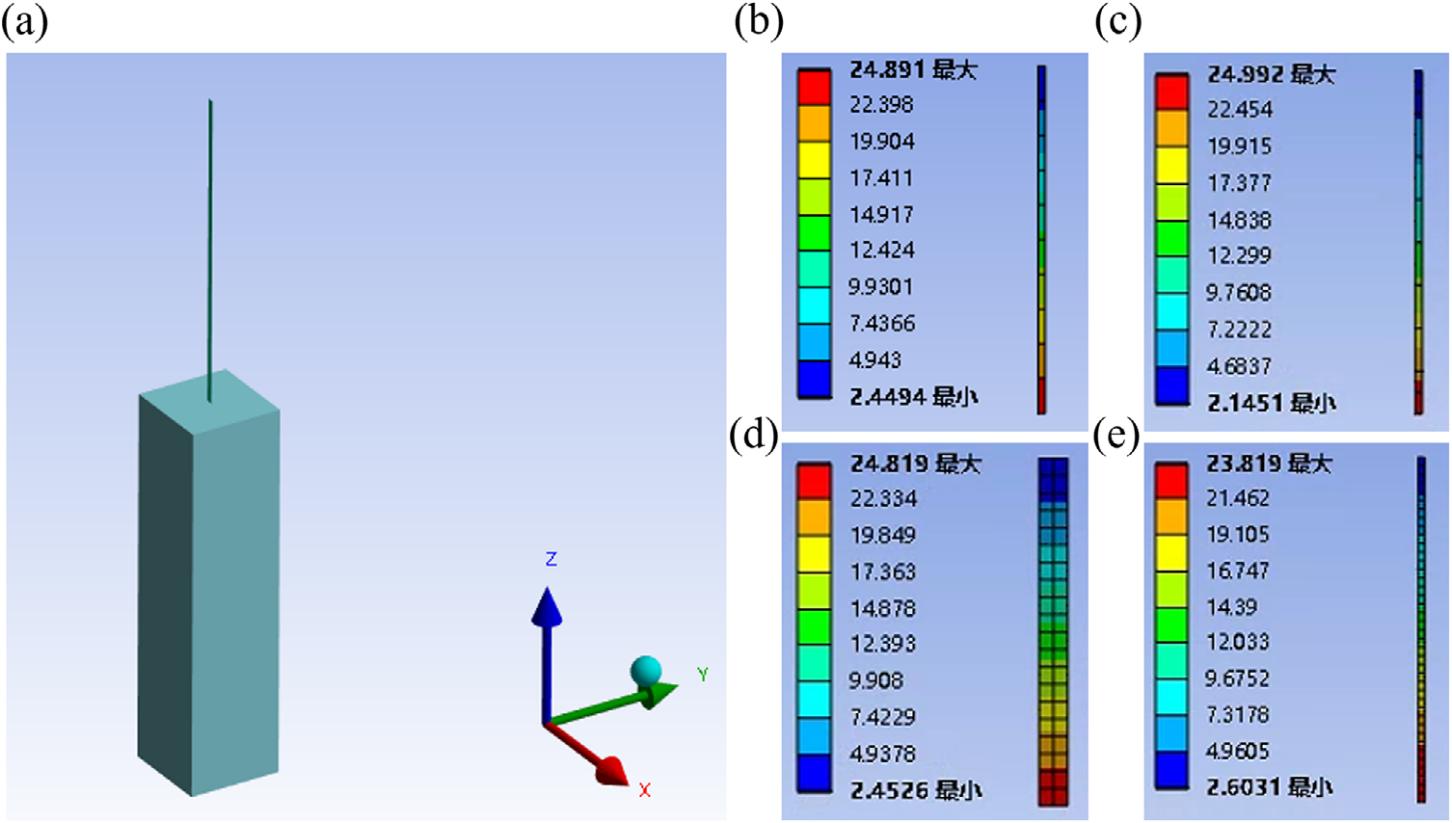
a) The finite element model of the electrode and the brain tissue. b)–e) The maximum amount of displacement at the interface between the electrodes of different sizes and the brain tissue.

A 3D finite element model of the electrode and brain tissue interface was developed in ANSYS WORKBENCH. In this study, it was assumed that the electrode was implanted in the brain. The full model of the electrode and the brain tissue was presented in **Figure** **2**a.

The brain tissue was 60 mm in length, 1.5 mm in width, and 1.5 mm in thickness. The original size of the electrode was 10 mm in length, 100 µm in width, and 3 µm in thickness. The implantation depth of the electrode was 5 mm. The brain tissue was considered as linear elastic and nearly incompressible for the static analysis.^[^ ^14^ ^]^ Since the damage area caused by the micromotion of the brain tissue was usually in the range of hundreds of micrometers. To limit sensitive areas, the distance between the boundary of the brain tissue and the electrode centerline was defined as 750 µm.^[^ ^15^ ^]^ All strain fields generated by the brain tissue micromotion were included to eliminate the influence of boundary effects.^[^ ^16^ ^]^ For the static analysis, the brain tissue was characterized by its Young's modulus of 6 kPa and its Poisson's ratio of 0.499.^[^ ^17^ ^]^ The electrode was characterized by its Young's modulus of 6 GPa and its Poisson's ratio of 0.33.^[^ ^18^ ^]^ One of the length, the width, and the thickness of the electrode were changed to observe the maximum displacement of the implanted end of the electrode. At the initial status of the simulation, the surfaces of the electrode and the brain tissue were in contact with each other. To simulate the micromotion, a displacement load was applied to the brain tissue and while the bottom of the brain tissue was fixed. For the convenience of research and analysis, the micromotion was only in the x direction and the micromotion amplitude was taken as 25 µm.^[^ ^19^ ^]^ It was demonstrated that the maximum displacement of the interface between the electrode and the brain tissue was 24.891 µm in the initial state in Figure 2b. With the increase of the length of the electrode, when the length of the electrode was 40 mm, the maximum displacement of the implanted end was 24.992 µm in Figure 2c. With the width or thickness of the electrode increased, the flexibility of the electrode became worse. When the width of the electrode was 400 µm, the maximum displacement was 24.819 µm in Figure 2d. When the thickness of the electrode increased to 12 µm, the maximum displacement at the interface was only 23.819 µm in Figure 2e. Considering the limitations of the manufacturing processes, the final dimensions of the electrode was determined to be 40 mm in length, 164 µm in width, and 3 µm in thickness.

## 3 Design and Fabrication of the Ultra‐Flexible Neural Electrode

Each electrode had 4 channels and the size of each eectrode recording point was 14 µm in length and 24 µm in width. The distance between the adjacent recording points was 500 µm and the width of the electrode connection wire was 10 µm. The implanted end of the electrode was designed to be like a pair of ears, which helped the electrode to stay stable in the nerve tissue after implantation. The width and the length for each ear were 20 and 123 µm respectively. There was a guide ring at the top of the implanted end to assist the surgical robot in implanting the electrode into a specific area or a specific location in the brain. The size of the guide ring was 140 µm in length and 40 µm in width. There was a large pad on the other end of the electrode, which measured 8.5 mm in length and 4.6 mm in width. Four large gold electrodes were distributed on this pad. They formed a stable electrical connection with the connector, and the signal was transmitted to the external device through the printed circuit board.

**Figure 3:**
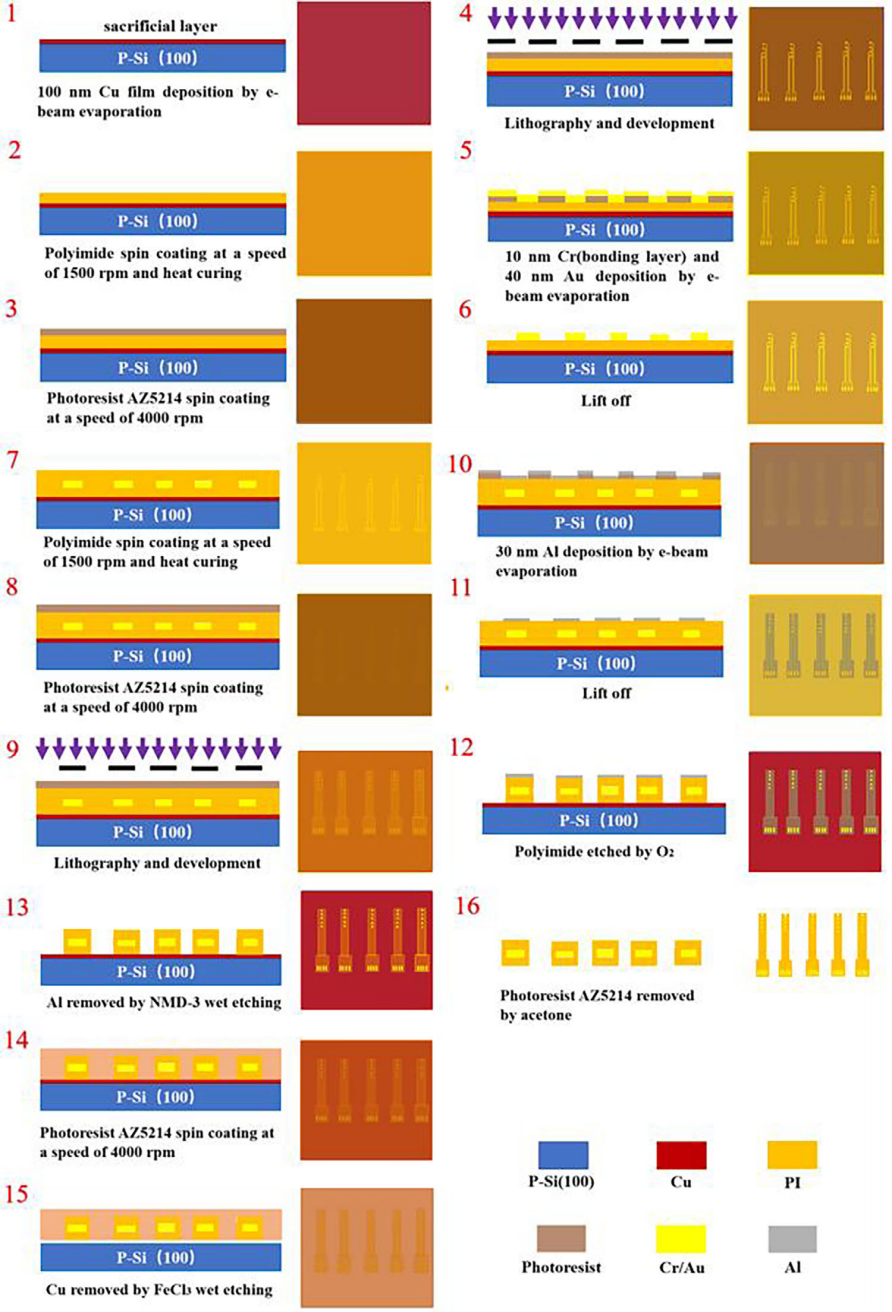
The fabrication process of the ultra‐flexible neural electrode.

**Figure 4:**
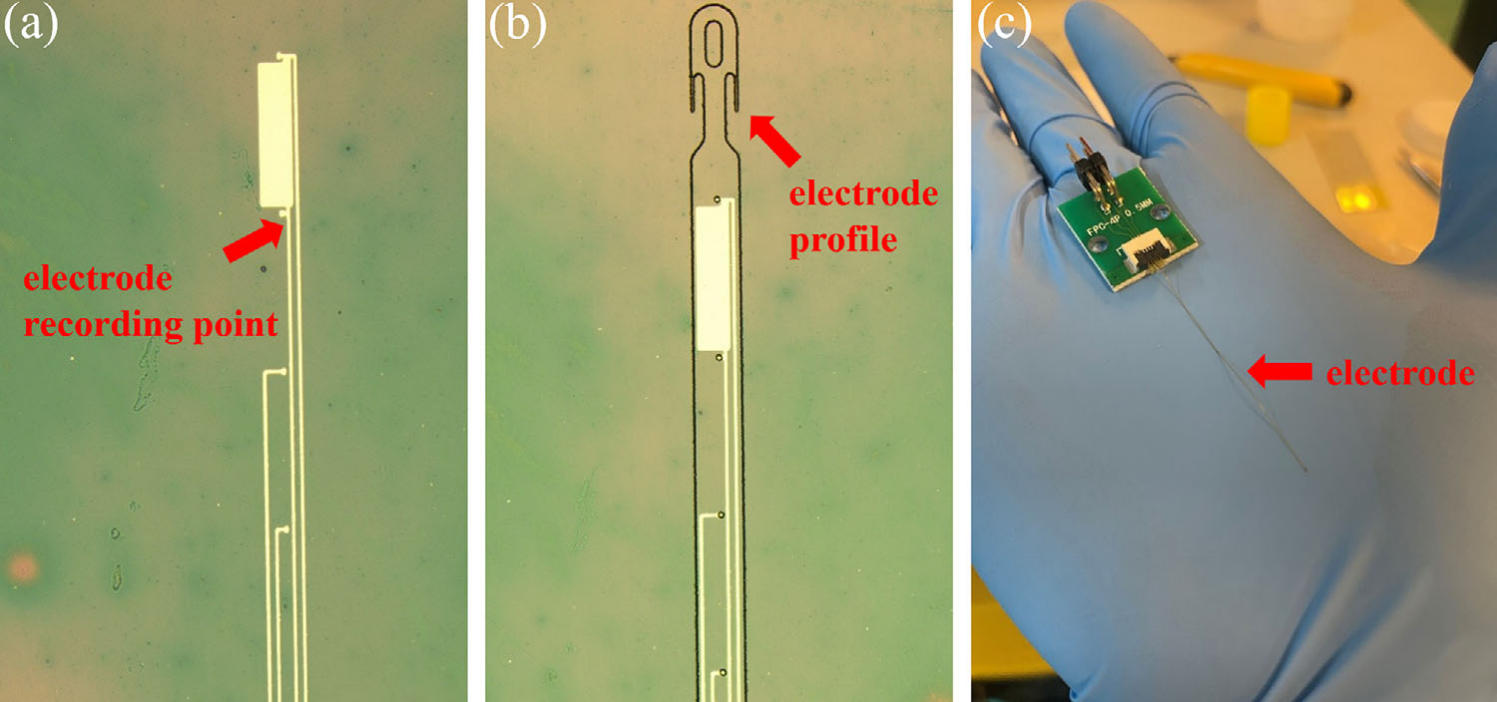
a) The recording points and the connection wires of the electrode. b) the whole profile of the electrode c) the assembled electrode.

**Figure** **3** shows the fabrication processes of the ultra‐flexible neural electrode. The silicon wafer was immersed in acetone for 10 min, rinsed with deionized water, and dried with nitrogen. A 100 nm copper film was deposited on the silicon substrate by electron beam evaporation as a sacrifice layer. The first polyimide layer was spin‐coated on the surface at a speed of 1500 rpm. The stress in the polyimide film was released as much as possible by step heating, and the polyimide film was finally cured with a thickness of ≈1.5 µm. A layer of photoresist AZ5214 was spin‐coated on the polyimide, and then the electrode recording points, the electrode connecting wires, and the large gold electrodes on the pad were patterned by ultraviolet lithography in **Figure** **4**a. 10 nm chromium film and 40 nm gold film were deposited by electron beam evaporation, in which chromium was used as the bonding layer to enhance the adhesion between the gold film and the polyimide layer. On this basis, the second polyimide layer was spin‐coated at a speed of 1500 rpm, and the polyimide film was cured by heating in the same way. Then, a layer of photoresist AZ5214 was spin‐coated on the surface of the electrode, followed by patterning the profile of the electrode, the masks of the electrode recording points, and the masks of the large electrodes on the pad by ultraviolet lithography. A 30 nm aluminum film was deposited on the surface of the electrode. When the extra aluminum film was removed with acetone, the remained aluminum film worked as the mask of the entire electrode except for the recording points and the electrodes on the pad. The polyimide layer outside the profile of the electrode, the polyimide layer of the electrode recording point surface, and the polyimide layer of the gold electrode surface on the pad were etched by O2(reactive ion etching). The profile of the electrode was completely displayed, and the electrode recording points and the electrodes on the pad were also completely exposed in Figure 4b. After etching, a layer of photoresist AZ5214 was spin‐coated on the entire electrode surface as a support layer, which was convenient for the electrode transfer. Finally, the wafer was placed in the ferric chloride solution. The sacrifice layer was gradually removed, and the electrode was totally released from the wafer eventually. The photoresist support layer was removed with acetone, cleaned with deionized water, and then naturally dried. After that, the electrode was connected to the flexible printed circuit in Figure 4c.

## 4 Characterization of Brain Micromotion Properties

**Figure 5:**
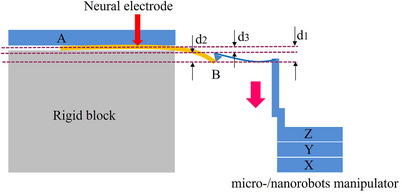
The theoretical model of the ultra‐flexible neural electrode.

For this ultra‐flexible neural electrode, it will be subjected to the force from brain tissue. This interaction force also reflects the compatibility between brain tissue and electrodes. Characterizing the mechanical property of the electrode with high precision in a conventional experimental environment was also a challenge. By combining the advantages of high vacuum and high resolution of the SEM and high manipulation accuracy of the micro‐/nanorobotic manipulation system, this problem could be solved. We assume a model of the electrode and brain tissue. The micromotion of brain tissue was simulated by the AFM cantilever. The top view of the theoretical model of the ultra‐flexible neural electrode and the AFM cantilever is shown in **Figure** **5**. The electrode was fixed on the side of the rigid block. Point A was the fixed end of the electrode, and point B was the implanted end of the electrode. The AFM cantilever (*k* = 0.03 N m^−1^) was opposite to the implanted end of the electrode. In this way, the tip of the AFM cantilever could press down the electrode in the direction perpendicular to the electrode. d1 was the displacement of the root of the AFM cantilever, which was the displacement of the manipulator. d2 was the displacement of the head of the AFM cantilever, which was the displacement of the electrode. d3 was the actual displacement of the AFM cantilever. According to Hooke's law,^[^ ^20^ ^]^ the interaction force between the electrode and the AFM cantilever was expressed as

(1) $$F=k\times \left(d_{1}-d_{2}\right)$$

where *k* was the spring constant of the AFM cantilever.

**Figure 6:**
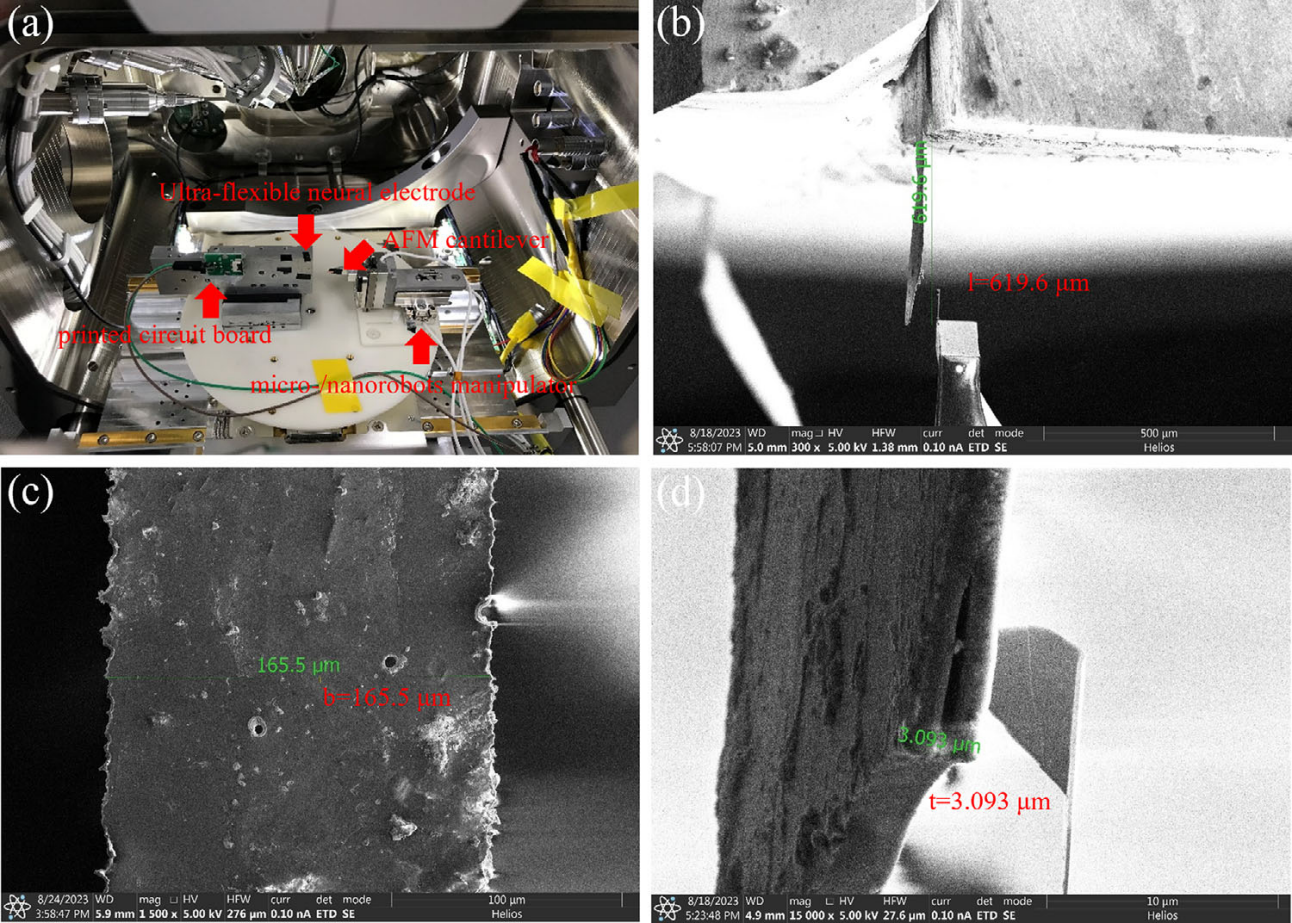
a) The micro‐/nanorobotic manipulation system under the SEM. b–d) The 3D size of the ultra‐flexible neural electrode.

**Figure 7:**
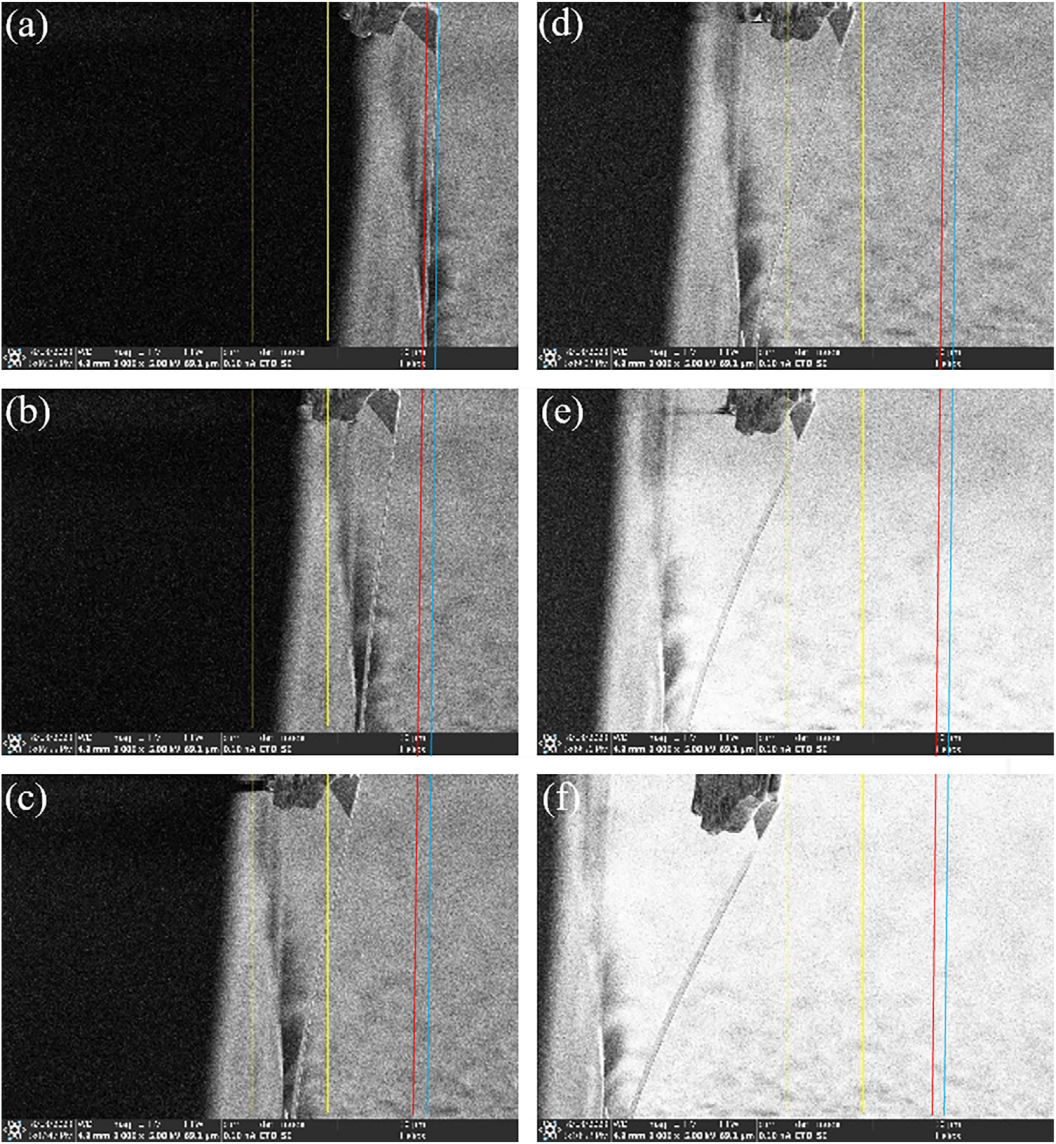
The downpressing process of the ultra‐flexible neural electrode. The blue line represented the original position of the head of the AFM cantilever. The red line represented the original position of the root of the AFM cantilever. The displacements from (a–f) were 0, 5, 10, 15, 20, and 25 µm, respectively.

**Figure** **6**a shows the micro‐/nanorobotic manipulation system under the SEM. The electrode and the printed circuit board with the connector were fixed on the side of the rigid block, and the opposite side of the electrode was the micro‐/nanorobotic manipulator, which could be driven by the actuator along the directions of x, y, and z. The AFM cantilever could apply the vertical displacement to the implanted end of the electrode. The external actuator and electrical test instrument (KEITHLEY 2634B) were connected to the internal experimental system through the sidewall of the chamber of the SEM. It was observed that the suspended length, the width, and the thickness of the electrode were 619.6, 165.5, and 3.093 µm, respectively, from Figure 6b–d. Then, the position of the AFM cantilever was adjusted to the same height as the implanted end of the electrode. After that, it gradually moved along the x direction until they contacted each other. In **Figure** **7**, the head of the implanted end of the electrode was pressed down by the tip of the AFM cantilever with the displacements of 5, 10, 15, 20, and 25 µm. The positions of the root of the AFM cantilever and the head of the implanted end of the electrode were recorded for different displacements.

**Figure 8:**
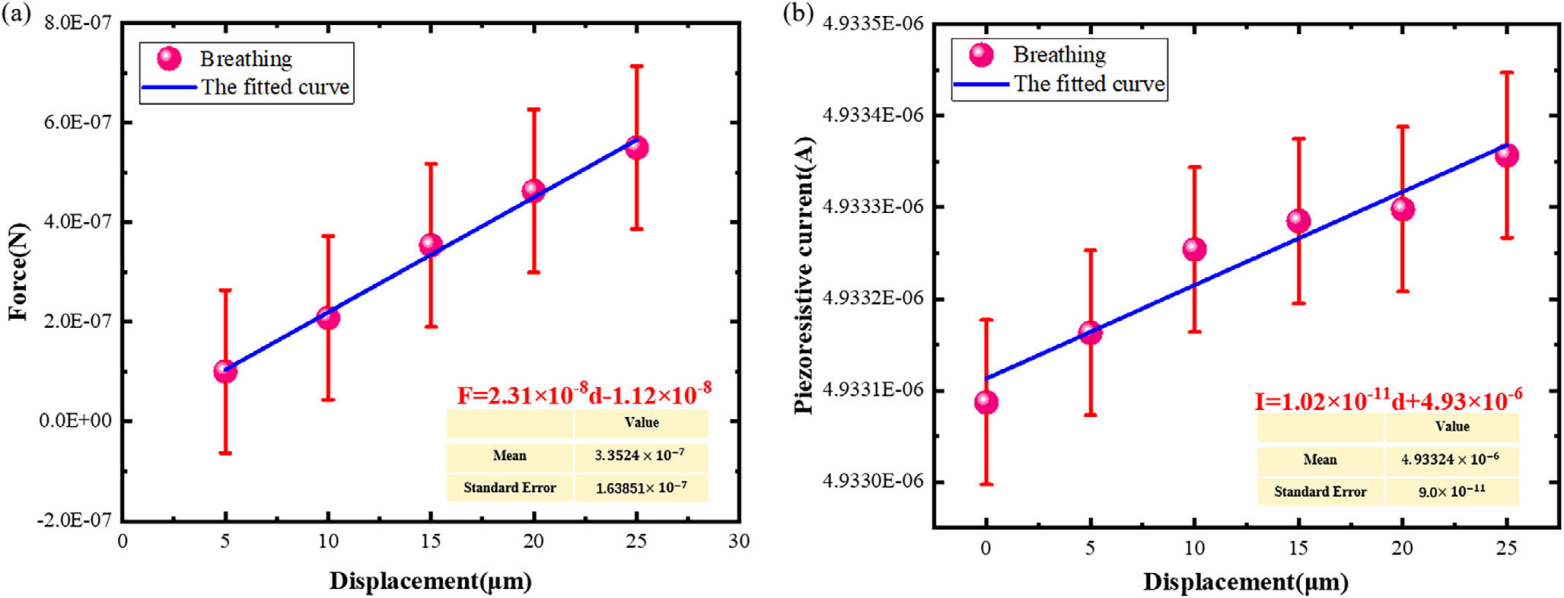
a) The relationship between the force and the displacement of the ultra‐flexible neural electrode in the downpressing process. b) The relationship between the piezoresistive current and the displacement of the ultra‐flexible neural electrode in the downpressing process.

The relationship between the displacement of the head of the implanted end and the corresponding interaction force was shown in **Figure** **8**a. The force applied on the electrode showed a linear relationship with the increase in head displacement. The equivalent stiffness of the electrode tip was 0.023 N m^−1^ by fitting the forces and the displacements under the five different displacements. According to the fitted relationship, the Young's modulus of the implanted end of the electrode was calculated to be 4.49 GPa, which was within the order of magnitude of conventional polyimide. The flexibility of the implanted end of the electrode was 4.33 *×* 10^7^ µm N^−1^. During the downpressing process, a constant voltage of 0.1 V was applied to the electrode to evaluate the piezoresistive characteristics for different displacements. Figure 8b shows the relationship between the piezoresistive currents and the displacements of the electrode. The piezoresistive current and the displacement showed a strong correlation in the whole deformation process. There was an obvious linear relationship between the piezoresistive current and the displacement of the head of the implanted end, which strongly indicated that the piezoresistive effect existed during bending deformation. Since the material of the conductive layer of the electrode was gold. The piezoresistive effect generally refers to the change in resistance due to the deformation of metals.

**Figure 9:**
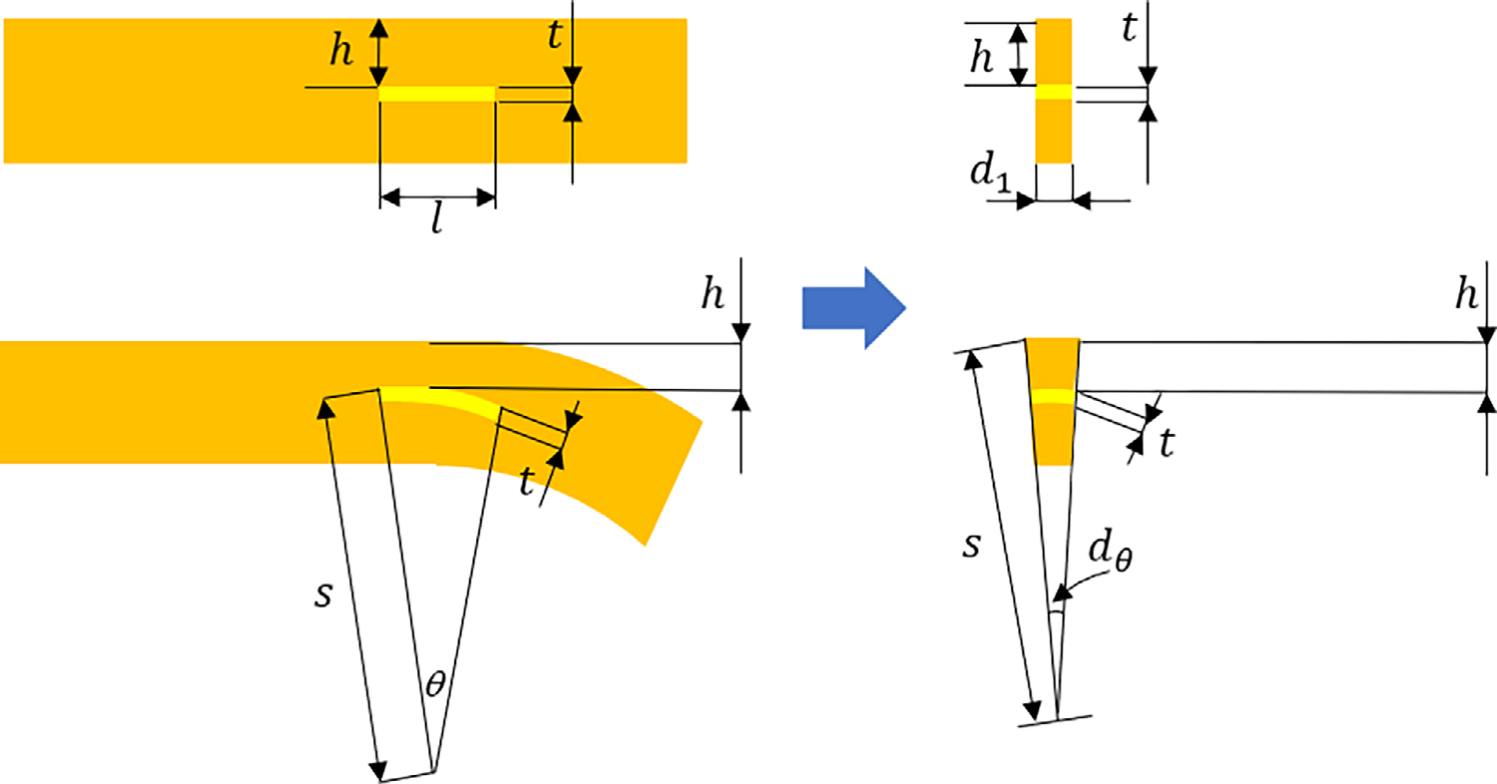
The schematic diagram of the electrode solid and the electrode element before and after the bending deformation of the ultra‐flexible neural electrode.

To better understand this mechanism, the theoretical model of the electrode before and after the bending deformation is shown in **Figure** **9**. Although the polyimide on the surface of the recording point was etched, most of the metal was still wrapped in the polyimide. Thus, the electrode model before and after the bending deformation could be simplified in this way.

In this model, *h* referred to the distance between the top gold film of the recording point and the above polyimide, *t* was the thickness of the recording point, *l* was the length of the electrode, *s* was the curvature radius of the bending, $d_{A_{1}}$ was the area of the initial cross‐section of the element of the electrode, and $d_{A_{2}}$ was the cross‐section of the element of the electrode after bending deformation. $d_{A_{1}}$ and $d_{A_{2}}$ could be expressed as:

(2) $$d_{A_{1}}=d_{1}\times t$$

(3) $${d_{A}}_{2}=\frac{1}{2}\;\left[2\times d_{l}\times t+\frac{d_{l}\times h^{2}}{s}-\frac{d_{l}\times {\left(h+t\right)}^{2}}{s}\right]\;$$

It was obvious that $d_{A_{2}}$ was smaller than $d_{A_{1}}$. While the thickness of the element was unchanged, the length of the electrode decreased after the bending deformation. According to the calculation equation of metal resistance, the length of the electrode decreased, and the resistance decreased accordingly. Thus, the piezoresistive current presented a linear upward trend. In addition, the sensitivity of the piezoresistive microdevice was one of the important performance indexes, which was defined by the ratio between the change of piezoresistive current and the change of the displacement at the head of the implanted end of the electrode. From the fitted curve, the sensitivity of the ultra‐flexible neural electrode was 10.2 pA µm^−1^.

## 5 Characterization of Impedance Property of the Ultra‐Flexible Neural Electrode

**Figure 10:**
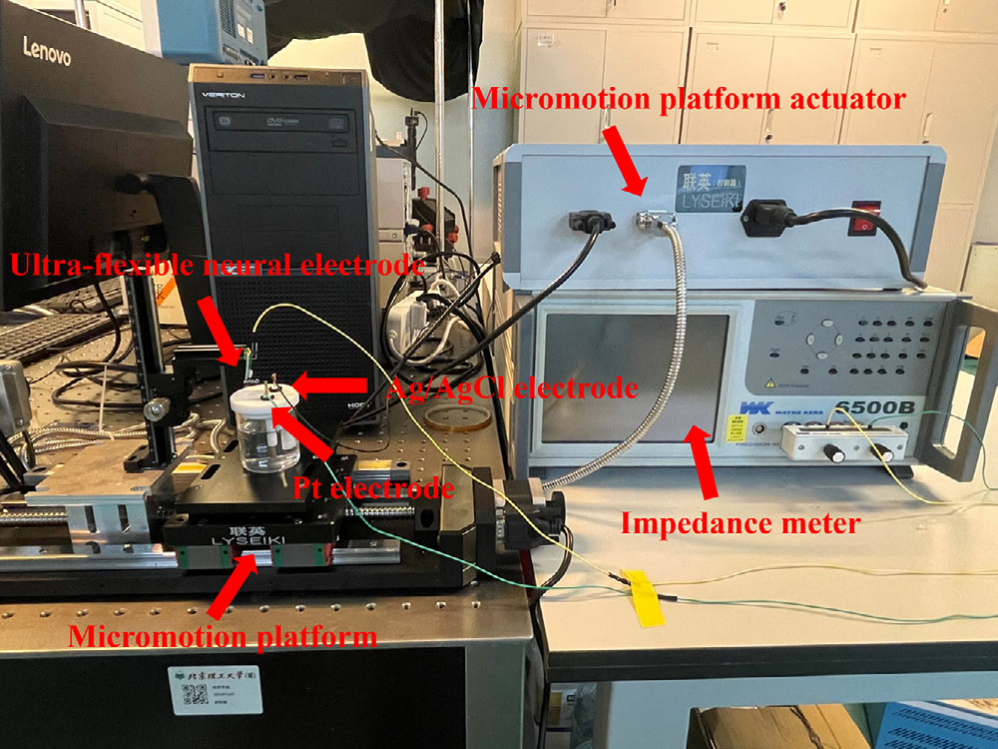
The schematic diagram of a micromotion platform for impedance characterization of the ultra‐flexible neural electrode.

To further understand the impedance characteristics of the ultra‐flexible neural electrode subjected to the brain tissue micromotion, a micromotion platform that simulated the micromotion of the brain tissue was built in **Figure** **10**.

In the three‐electrode system, the ultra‐flexible neural electrode and the Ag/AgCl electrode served as the working electrode and the reference electrode, respectively. The platinum electrode worked as the counter electrode, and the size was 10 mm in length, 10 mm in width, and 0.1 mm in thickness. The area was much larger than that of the working electrode to reduce the influence of the auxiliary electrode polarization on the working electrode. The three‐electrode system was fixed on the micromotion platform, and the motion accuracy of the platform was 1 µm. The applied AC voltage was 10 mV and the frequency range was from 20 Hz to 500 kHz. First, the impedance of the working electrode was recorded in the 0.01 mol l^−1^ Phosphate Buffered Saline (PBS) solution when the platform was in a static state. In the following procedures, the displacements of the platform were set to 5, 10, 15, 20, and 25 µm. The corresponding impedances of the working electrode under different displacements were also simultaneously recorded.

**Figure 11:**
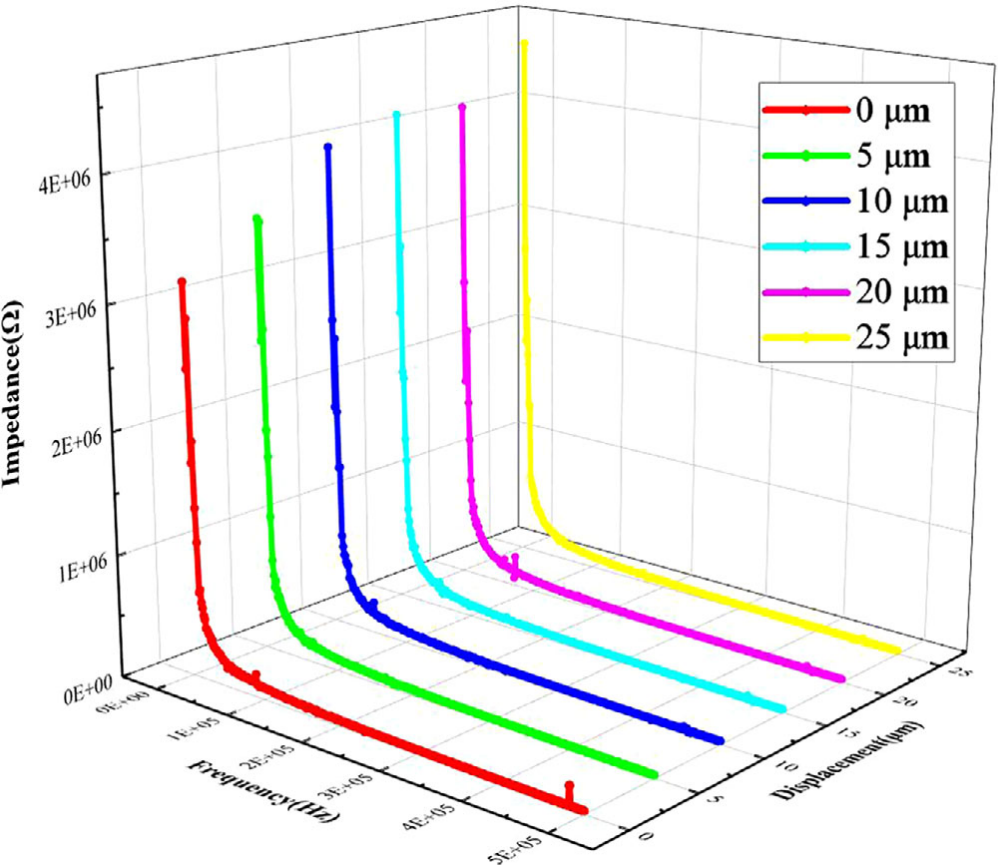
The impedance spectrum of the electrode‐PBS solution interface under different displacements.

**Figure 12:**
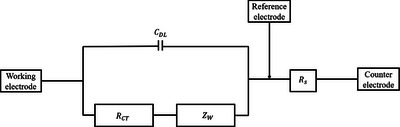
The equivalent circuit of the electrode‐PBS solution interface impedance.

**Figure** **11** shows the impedance spectrum of the working electrode under the different displacements. For the same displacement, the impedance of the electrode decreased with the increase of frequency. The impedance characteristic of the electrode at 1 kHz was a typical value. The initial impedance at 1 kHz was 3.17 MΩ and the remaining five groups of impedances at 1 kHz were measured at 3.53, 3.97, 4.12, 4.07, and 4.51 MΩ with the increase of the displacement. It was obvious that the impedances showed an increasing trend with the increasing displacements. To explain this phenomenon, the interface impedance equivalent circuit between the electrode and the PBS solution is shown in **Figure** **12**.

The equivalent interface impedances included the double‐layer capacitance (*C_DL_*), the charge conduction resistance (*R_CT_*), Warburg impedance (*Z*_ω_), the electrode wires and PBS solution resistance (*R_s_*). Warburg impedance reflected the effect of reactant migration on charge conduction and could be expressed as

(4) $$Z_{W}=\sigma \times \frac{1}{{\left(j\omega \right)}^{1/2}}$$

At high frequencies, *Z*_ω_ was much smaller than *R_CT_*. Thus, the impedance could be expressed as

(5) $$Z_{HF}=R_{s}+R_{CT}\times \frac{1-j\omega C_{DL}R_{CT}}{1+{\left(\omega C_{DL}R_{CT}\right)}^{2}}$$

Since *C_DL_* was proportional to the area of the electrode and *R_CT_* was inversely proportional to the area of the electrode, the impedance could also be expressed as

(6) $$Z_{HF}=R_{s}+\frac{\rho l}{A}\times \frac{1-j\omega \varepsilon \rho }{1+{\left(\omega \varepsilon \rho \right)}^{2}}$$

Therefore, the impedance of the electrode at 1 kHz could be approximately considered to be inversely proportional to the area of the electrode. According to the previous analysis, the area of the electrode decreased after bending deformation. The impedance of the electrode showed an upward trend during the downpressing process.

## 6 Electrophysiological Recordings of the Ultra‐Flexible Neural Electrode

**Figure 13:**
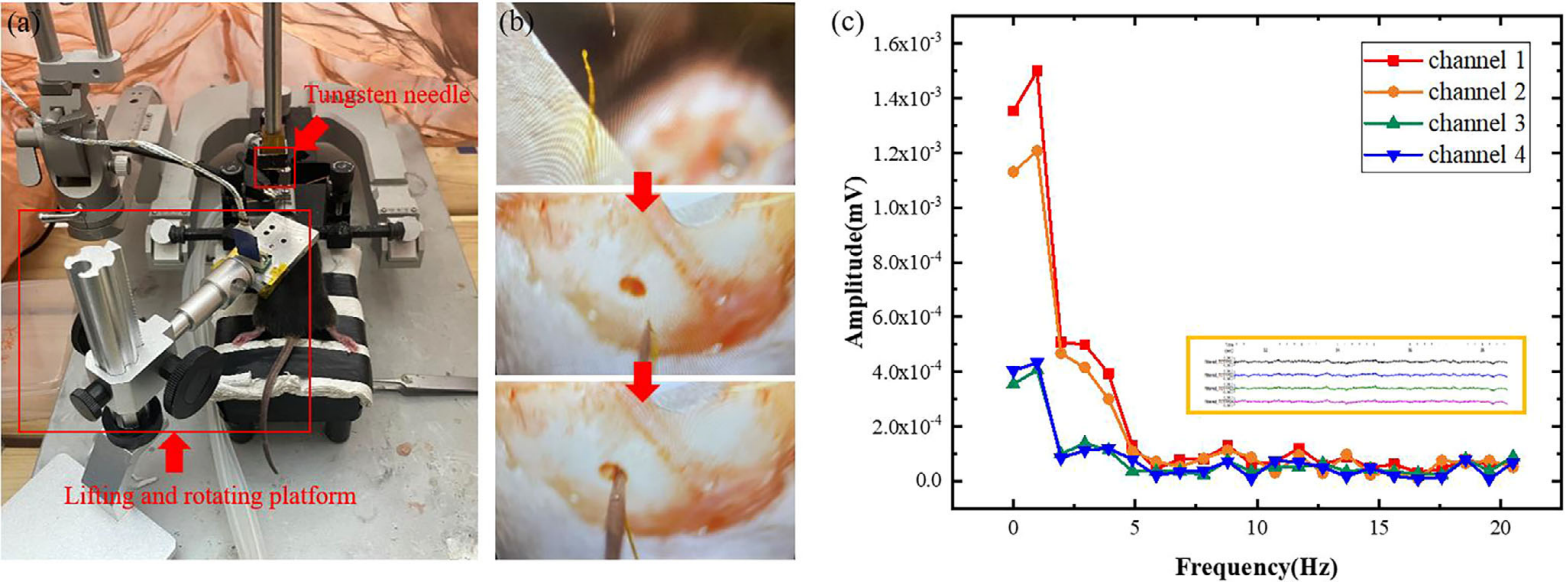
The electrophysiological recordings of the ultra‐flexible neural electrode. a) The electrode implantation platform. b) The process of electrode implantation. c) The recorded local field potential signal amplitudes after the Fourier Transform. The recorded local field potential signals are shown in the orange box.

The C57BL/6J mouse was used in electrophysiological recording experiments. The animal care and experimental protocols were approved by the Animals Research Ethics Committee of the Shanghai University of Traditional Chinese Medicine. The electrode implantation experimental platform for mice is shown in **Figure** **13**a. The mouse is fixed on a stereotaxic instrument, where anesthesia and craniotomy are performed to expose the target brain region for electrode implantation. The equipment connected to electrodes is placed on a lifting and rotating platform, which allows height and angular adjustments to ensure the electrode implantation segment aligns precisely above the target brain area while preventing electrode breakage caused by tension during implantation. A tungsten needle is fixed to the stereotaxic instrument. Figure 13b illustrates the electrode implantation process: The tungsten needle passes through the guidance ring at the electrode implantation end to position the electrode directly above the target brain region. The electrode is then manually implanted to a depth of 3 mm using the vertical rod knob on the stereotaxic instrument. We employed Plexon Inc.’s 96‐channel in vivo recording system from the United States. After implantation, local field potentials from neural tissues were successfully recorded, as demonstrated in the orange box in Figure 13c. A Fourier transform is performed on the recorded local field potentials (LFPs) to convert the time domain signal into the frequency‐domain signal. According to the documents of Xi'an Jiaotong University Laboratory Animal Center, the breathing frequency of a mouse ≈2.72 Hz (1.4–3.83 Hz). we converted the acquired neural signals into frequency‐domain data and extracted this part of information of 1–20 Hz. Theoretically, there is no significant difference in the amplitude of signals in mice in a similar frequency range. In contrast, the signal spectrum analysis revealed the signal intensities close to 2.72 Hz were significantly higher than those of 5–20 Hz for each electrode channel. Since the positions of recording point 1 and recording point 2 are closer to the head of the electrode implantation end, the signal amplitude of channel 1 and channel 2 is significantly enhanced compared to channel 3 and channel 4. These findings suggest that this electrode achieves excellent mechanical compatibility with brain tissue, demonstrating distinct advantages in recording neural signals.

## 7 Conclusion

This study addresses the core issue of insufficient mechanical compatibility between implantable electrodes and brain tissue by proposing an innovative design framework based on the synergistic regulation of materials and structures. It reveals the active compensation mechanism of electrode deformation at the exposed end for brain tissue micromotion, successfully achieving zero relative motion at the implantation interface. A quantitative design system for mechanical matching between electrodes and brain tissue has been established, providing a new paradigm for electrode design and long‐term stable neural signal acquisition. The developed dual‐mode electrode integrates both neural signal acquisition and micromotion sensing capabilities, breaking through the technical bottleneck of single‐functionality in traditional devices and laying the foundation for hardware implementation of closed‐loop brain‐computer interfaces. Furthermore, through innovative integration of the micro‐/nanorobotic manipulation system and bionic brain environment simulation technology, dynamic synchronous detection of electrode mechanical characteristics (A displacement of 25 µm corresponds to a force of 575 nN) and electrical properties (6.4 pA mm^−1^ length sensitivity and 10.2 pA µm^−1^ displacement sensitivity) has been achieved. The experimental results demonstrate that the optimized electrode not only enables stable electrophysiological signal acquisition but also is expected to detect brain tissue micromotion via piezoresistive responses, paving the way for multifunctional neural interfaces. This work provides a theoretical framework and technological prototype for next‐generation BCI devices, holding significant clinical potential.

## Conflict of Interest

The authors declare no conflict of interest.

## Data Availability

The data that support the findings of this study are available from the corresponding author upon reasonable request.
